# Anatomical Variations in Major Abdominal Aortic Branches and Sex-Related Differences: A Large-Scale Analysis of 1174 Patients

**DOI:** 10.3390/tomography12040051

**Published:** 2026-04-06

**Authors:** Oguzhan Tokur, Koray Bingol

**Affiliations:** 1 Department of Radiology, Kütahya Health Sciences University, Kütahya 43100, Türkiye; 2 Department of Anatomy, Erzincan University, Erzincan 24002, Türkiye; koray.bingol@erzincan.edu.tr

**Keywords:** abdominal aorta, anatomical variations, computed tomography angiography, sex-related differences

## Abstract

Background: This study aims to evaluate the prevalence, spectrum, and coexistence of anatomical variations in the major branches of the abdominal aorta using Multidetector Computed Tomography (MDCT) angiography, with a specific emphasis on analyzing sex-related differences in a large-scale cohort. Methods: A retrospective analysis was conducted on 1174 patients (63.8% male, 36.2% female; mean age 60.54) who underwent abdominal CT angiography between January 2023 and June 2024. Images were acquired using a 128-slice MDCT scanner and reconstructed for detailed vascular assessment. Statistical comparisons between genders were performed using Chi-square and Fisher–Freeman–Halton tests, with *p* < 0.05 considered significant. Results: The celiac trunk (93.3%), superior mesenteric artery (SMA) (97.1%), and inferior mesenteric artery (IMA) (98.5%) predominantly showed classical patterns. However, significant sex-related differences were identified. Females exhibited significantly higher rates of classical patterns for the celiac trunk (96.2% vs. 91.7%), IMA (99.1% vs. 98.1%), right hepatic artery (RHA) (91.5% vs. 82.6%), and left hepatic artery (LHA) (95.8% vs. 85.4%). Conversely, males showed a higher prevalence of complex variations, including replaced/accessory hepatic arteries and the absence of the common hepatic artery. The number of right and left renal arteries was similar between sexes and did not show a significant difference, while horseshoe kidney was detected only in males. Conclusions: Abdominal vascular structures adhere to classical anatomy more frequently in females, while males exhibit greater morphological variability. These findings emphasize the necessity of gender-specific preoperative vascular mapping to optimize surgical outcomes and reduce morbidity.

## Simple Summary

This study investigates the differences in major arterial branches of the abdominal aorta between male and female patients. While most people follow standard patterns, our research shows that female individuals are more likely to have “classic” anatomy, whereas male individuals display more complex variations, particularly in vessels leading to the liver and kidneys. These findings suggest that surgeons and radiologists should consider a patient’s gender when planning procedures to avoid complications. By highlighting these differences, this study promotes more personalized medical imaging and surgical approaches, ensuring safer outcomes and paving the way for further research into anatomical diversity.

## 1. Introduction

The abdominal aorta is the segment of the aorta extending between the T12 and L4 vertebral levels, from which major arteries—including the celiac artery, superior mesenteric artery (SMA), inferior mesenteric artery (IMA), and renal arteries—originate. Variations arising during embryologic development can result in a wide spectrum of morphological differences in these branches.

In the classic anatomical arrangement, the celiac trunk typically presents as a normal trifurcation, dividing into the left gastric artery (LGA), splenic artery (SA), and common hepatic artery (CHA). The superior mesenteric artery (SMA) and inferior mesenteric artery (IMA) conventionally arise as independent anterior branches from the abdominal aorta. For the renal vasculature, the standard morphology consists of a single right and a single left renal artery originating directly from the lateral aspects of the abdominal aorta. Furthermore, in the classic hepatic arterial configuration, both the right hepatic artery (RHA) and left hepatic artery (LHA) originate from the proper hepatic artery. Establishing these standard morphologies is essential for recognizing anatomical variations, which can significantly alter preoperative planning and surgical outcomes. Although the classic branching pattern of the major vessels is well established, awareness of anatomic variations is particularly important prior to surgical and interventional procedures.

Preoperative vascular mapping has become a crucial requirement for the effectiveness and safety of surgical procedures. The literature indicates that serious complications due to injury to a previously unknown vascular variation during the procedure lead to a significant increase in morbidity and mortality [1,2,3]. Therefore, preoperative vascular mapping has become an essential requirement, especially in oncological surgeries, organ transplantations, and interventional radiological procedures such as transcatheter arterial chemoembolization (TACE) and transcatheter arterial radioembolization (TARE) [4,5,6].

Initially, vascular variations arising from the abdominal aorta were demonstrated through small-scale cadaver studies or from data obtained intraoperatively [7,8,9]. Currently, however, radiological imaging techniques such as MDCT enable rapid, non-invasive, and detailed evaluation in large cohorts. In particular, MDCT angiography (CTA) is considered the gold standard among non-invasive vascular imaging methods [10].

Although CTA-based studies in the literature demonstrated abdominal aortic variations, most are limited by relatively small cohorts or the absence of a comprehensive assessment of sex-related differences [11,12,13,14,15,16]. Therefore, the aim of this study was to evaluate the prevalence, spectrum, and coexistence of anatomical variations in the major abdominal aortic branches using MDCT angiography in a large-scale cohort of 1174 patients, with a specific focus on analyzing sex-related differences.

## 2. Materials and Methods

This retrospective study was conducted in our hospital between January 2023 and June 2024 and received approval from the Binali Yıldırım University Clinical Research Ethics Committee (Approval Date: 6 March 2023, Number: 2023-3/5). Routine consent was obtained from all patients before imaging examinations as part of standard clinical practice. However, because of the retrospective design of the study, the requirement for additional informed consent for participation was waived by the local ethics committee.

The abdominal aorta CT angiography images were acquired with a 128-slice MDCT scanner (Somatom go.Top, Siemens Healthcare, Forchheim, Germany). An 80 kVp dose was consistently maintained in all patients using a standard imaging protocol, and the mAs (180–220) was automatically set by the device based on the patient’s weight. During the procedure, 1.5−2 mL/kg of non-ionic contrast agent was injected intravenously at a rate of 4 mL/sec via an automatic injector system, followed by a saline solution. The scan encompassed the area extending from 2 cm above the diaphragm to 4–5 cm below the level of the symphysis pubis. The arterial phase images were obtained at an average of 20–30 s, once the ROI placed on the proximal abdominal aorta reached a threshold of 150 HU. Initially, the images were acquired with a 2 mm slice thickness in the axial plane and were later reconstructed at a slice thickness of 0.625 mm. As the thin-slice axial images were analyzed by a single seven years-experienced radiologist specializing in abdominal imaging on the workstation, reformats in the coronal and sagittal planes were created, and 3D reconstructions were generated using the volume rendering technique. Sectional images were analyzed using the PACS system (Akgun PACS Viewer v7.5; Akgün Software, Ankara, Turkey).

**Figure 1:**
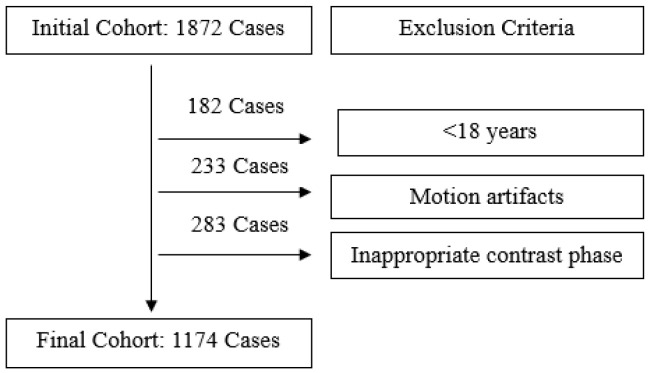
Study population and exclusion criteria.

Patients under 18 years of age, and those with significant motion artifacts or images where arterial structures could not be optimally assessed due to inappropriate contrast phases, were excluded from the study. The selection process for the study population and the specific exclusion criteria are detailed in Figure 1.

## 3. Statistical Analysis

Descriptive statistics for continuous variables were expressed as mean ± standard deviation (SD) and [minimum–maximum] values, while categorical variables were reported as frequencies and percentages. The normality of data distribution was assessed using the Shapiro–Wilk test. For the comparison of two independent groups, the Student’s *t*-test was used for normally distributed variables, and the Mann–Whitney U test was used for non-normally distributed variables. Associations between categorical variables were analyzed using the Pearson Chi-square test or the Fisher–Freeman–Halton Exact Test when expected frequencies were low. All statistical tests were two-tailed, and a *p* value of <0.05 was considered statistically significant. Data coding, cleaning, and statistical analyses were performed using IBM SPSS Statistics for Windows, Version 29.0 (IBM Corp., Armonk, NY, USA).

## 4. Results

A total of 1174 cases were evaluated in this study. Of the patients, 63.8% were male and 36.2% were female, with a mean age of 60.54 ± 15.43 years (range: 21–92 years) (Table 1). The origin of the celiac trunk was consistent with the classic anatomical pattern in the majority of cases (93.3%). The most frequently observed variations were the gastrosplenic trunk (3.2%) and the hepatosplenic trunk (2.4%). The celiacomesenteric trunk and the absence of the celiac trunk were quite rare (0.3% each). Regarding the origin of the celiac trunk branches, the most common variations were the left gastric artery (LGA) arising from the abdominal aorta (0.7%), the LGA arising from the splenic artery (0.7%), and the absence of the common hepatic artery (CHA) (1.5%). Complex or multiple variations were rarely observed (0.3% each) (Table 1).

The origin of the superior mesenteric artery (SMA) was mostly classical (97.1%). The hepatomesenteric trunk (1.7%) and bimesenteric trunk (0.5%) were the most commonly observed variations. When evaluating variations in SMA branches, the replaced right hepatic artery (RHA) was the most frequent variation, observed in 10.0% of the participants. Other variations had a low prevalence, and SMA branches showed a classical distribution in 87.9% of cases (Table 2).

The inferior mesenteric artery had a classical origin in the vast majority of cases (98.5%). A bimesenteric trunk (0.5%) and origin from the SMA (0.3%) were rarely detected variations. The mean number of right renal arteries was 1.10 ± 0.31 (range: 1–3), and the mean number of left renal arteries was 1.14 ± 0.38 (range: 1–3). Renal anomalies were quite rare; the most common variant was rotational anomaly (1.0%), and no additional renal anomaly was present in most individuals (98.6%) (Table 2).

The origin of the right hepatic artery largely showed a classical pattern (85.8%). The most common variation was the replaced RHA originating from the SMA (9.7%). Accessory hepatic arteries were infrequent, with variation rates generally below 1%. The left hepatic artery (LHA) was also mostly in the classical location (89.1%). The most frequent variations were a replaced (5.5%) or accessory (4.7%) LHA originating from the LGA. An LHA originating from the SMA or the celiac trunk was quite rare (0.3% each) (Table 3).

When the distribution of vascular variations according to sex was evaluated, the classical celiac trunk pattern was observed at a significantly higher rate in females (96.2%) compared to males (91.7%) (*p* = 0.028). The classical origin of the celiac trunk branches was also observed more frequently in females. In the male sex, variations such as the absence of the CHA, arising from the abdominal aorta (AA), and the left gastric artery originating from the splenic artery (SA) or AA were found to occur at higher rates (*p* < 0.001) (Table 4).

The classical origin of the IMA was observed at a statistically significantly higher rate in females (99.1%) compared to males (98.1%). Furthermore, bimesenteric trunks and indistinguishable origins were detected more frequently in males, whereas an IMA originating from the SMA was rarely seen in females (0.9%) (*p* < 0.001) (Table 5).

The distribution of renal anomalies showed a distinct difference regarding sex (*p* < 0.001). The rate of renal rotational anomaly was 2.8% in females, while it was not observed in males. Conversely, horseshoe kidney was detected only in males (0.5%) (Table 6).

The number of right and left renal arteries was similar between sex and did not show a significant difference (*p* = 0.078 and 0.093, respectively).

**Figure 2:**
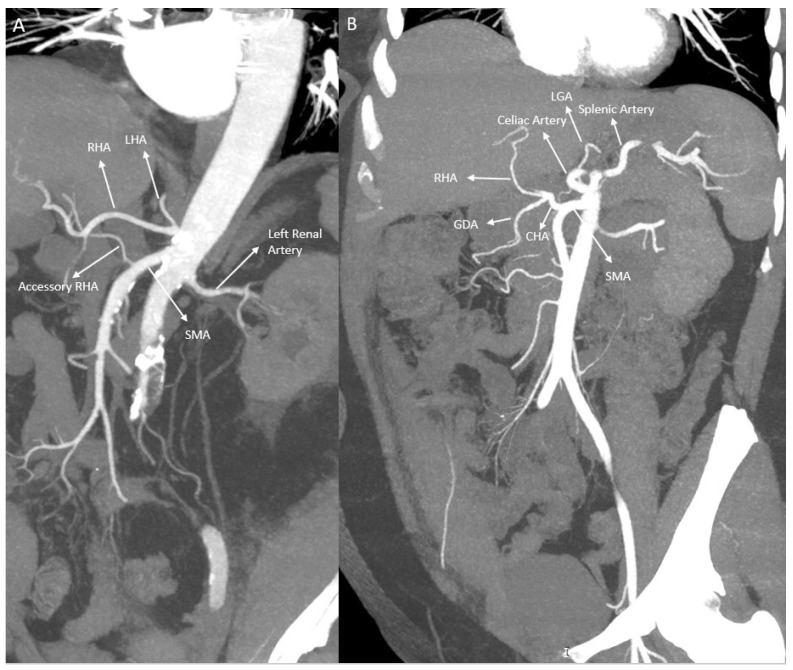
Anatomical variations in the hepatic arteries in two different patients: (**A**) Accessory right hepatic artery (RHA) and (**B**) common hepatic artery (CHA) originating from the superior mesenteric artery (SMA). LHA: left hepatic artery, LGA: left gastric artery, GDA: gastroduodenal artery.

**Figure 3:**
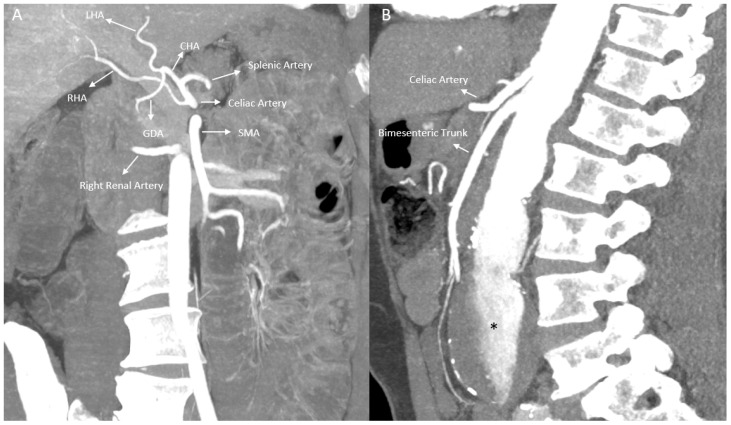
(**A**) Replaced RHA originating from the celiac trunk and (**B**) bimesenteric trunk in different patients. RHA: right hepatic artery, LHA: left hepatic artery, SMA: superior mesenteric artery, GDA: gastroduodenal artery, CHA: common hepatic artery. * Note a fusiform aneurism in abdominal aorta.

Regarding the RHA, the classical origin was observed at a statistically significantly higher rate in females (91.5%) (*p* < 0.001). Replaced RHA (especially originating from the SMA) and accessory hepatic artery variations were seen more frequently in males. In females, replaced arteries originating from the gastroduodenal artery (GDA) or LHA were detected at low rates (Figure 2 and Figure 3) (Table 6).

The LHA showed a classical origin in nearly all females (95.8%), which was a statistically significantly higher rate compared to that observed in males (85.4%) (*p* < 0.001). Replaced and accessory left hepatic arteries originating from the LGA were notably more common in males.

The SMA origin showed a similar distribution between sex and the difference was not statistically significant (*p* = 0.100) (Table 5).

## 5. Discussion

During embryonic development, the ventral splanchnic arteries and dorsal aortae fuse along the midline and form anastomotic networks, whereas certain primitive branches regress. This complex remodeling process determines the final vascular branching pattern, and vascular variations are thought to result primarily from incomplete or aberrant fusion, persistence of normally regressing vessels, or failure of the usual regression pathways. Several cellular factors and local molecular interactions are known to influence vasculogenesis and angiogenesis during this period. Genetic and ethnic factors may also play a role in vascular development. Although sex-related differences were observed in our study, no clear pathogenetic mechanism has yet been established to explain these findings [17,18,19,20,21].

Our study demonstrates substantial sex-based differences in the distribution of abdominal vascular variations, especially in regard to the renal artery, IMA, and celiac trunk. Classical-type aortic variations are significantly more common in women than in men. In particular, 96.2% of females and 91.7% of males had the classic celiac trunk type (*p* = 0.028), and the female population also had considerably higher rates of classical origins for the left and right hepatic arteries (*p* < 0.001). On the other hand, male individuals demonstrated a higher propensity for complex arterial variants. These included a higher incidence of the common hepatic artery arising directly from the abdominal aorta, as well as replaced and accessory hepatic arteries originating from the SMA or left gastric artery LGA (*p* < 0.001). In addition, the renal arteries demonstrated significant sex-related differences in morphology (*p* < 0.001) but the median number of renal arteries was similar in both genders, indicating no statistical difference between male and female patients.

In our study, the classic trifurcation pattern of the celiac trunk was observed in 93.3% of cases. This prevalence is significantly higher than the 68.0% observed in a Vietnamese population by Ngo Xuan Khoa et al. [16] and the pooled prevalence of 83.39% reported in a recent meta-analysis by Triantafyllou et al. [22]. However, our findings are consistent with other studies on the Turkish population, such as Bingol et al. (92.0%) [11] and Gümüş et al. (91.7%) [14], suggesting that regional or ethnic factors may influence these anatomical distributions. Between the non-classic types, the gastrosplenic trunk was the most frequent celiac trunk variation in our cohort (3.2%), which is similar to the study of Jalamneh et al. [15].

In contrast to many previous reports, we aimed to comprehensively characterize sex- related differences in vascular variations. Our results demonstrate that female individuals exhibited significantly higher incidence of classic arterial patterns compared to males; specifically, the classic celiac trunk configuration was found in 96.2% of females versus 91.7% of males (*p* = 0.028). Conversely, male individuals exhibited a higher incidence for complex variations, such as the absence of the common hepatic artery or its independent origin from the abdominal aorta. Góes Junior et al. [13] reported in the study that males tend to have larger arterial diameters and more acute origin angles. Additionally, our study also revealed that male individuals are more likely to have abdominal vascular variations. This suggests that sex-related differentiation should be taken into account in preoperative risk assessments for abdominal interventions.

Hepatic arterial system variations were frequently encountered and have substantial surgical relevance such as pancreatoduodenectomy and liver transplantation. Replaced RHA variation, originating from the SMA, was the most common variation in our study, observed in 10.0% of individuals. This finding was consistent with Gümüş et al. (10.1%) [14] and Dabria et al. [12] (13.6%), although it was slightly higher than that reported by Bingol et al. (8.0%) [11]. Notably, our sex-related analysis revealed that this specific variation was nearly 3.5 times more common in males (13.1%) than in females (3.8%). Awareness of RHA during surgical operations, such as liver transplantation and Whipple procedures, is critical to prevent iatrogenic right hepatic lobe injury; therefore, preoperative vascular mapping with MDCT angiography is essential for these operations.

SMA and IMA demonstrated significant anatomical stability in our study. The classic SMA origin was identified in 97.1% of cases, which is highly consistent with the results of Khoa et al. (96.3%) [16] and Bingol et al. (95.5%) [11]. Similarly, IMA exhibited a classic aortic origin in 98.5% of our cohort, consistent with the 98.4% reported by Bingol et al. [11]. Although they are relatively uncommon, variants like the celiacomesenteric trunk or the IMA arising from the SMA (reported in 2.6% of the Vietnamese population but only 0.3% in our cohort) are surgically important for colorectal surgeons to recognize in order to avoid impaired perfusion of the colon during resections.

In the study, we also compared sex-related differences in the number of renal arteries; the overall median number was 1 on both right and left side and no statistically significant difference was observed (*p* = 0.078 and 0.093, respectively). In contrast, Jalamneh et al. [15] and Kornafel et al. [23] revealed that renal anomalies, such as accessory arteries, were more frequent in males and more prevalent on the left side. This discrepancy might be attributed to variations in sample size, statistical power, or the specific demographic and ethnic characteristics of our study population compared to those in the cited literature.

## 6. Limitations

This study has several limitations. Firstly, its retrospective, single-center design may cause selection bias. In addition, clinical outcome data for the identified variations were not available for majority of patients. Future prospective studies correlating these anatomical findings with long-term clinical or surgical results would be beneficial. Secondly, in our study, the imaging data were evaluated by a single observer. Although the evaluations were conducted by an experienced radiologist according to established classification systems, the lack of interobserver agreement analysis should be taken into consideration when interpreting the results. Thirdly, our results may not be generalizable to other ethnic groups since the study was conducted in the Turkish population. Finally, we did not evaluate the direct impact of these variations on clinical outcomes, surgical success rates, or long-term complications. Future multicenter, prospective studies would be valuable to confirm these findings and clarify their practical relevance.

## 7. Conclusions

Vascular variations in the branches of the abdominal aorta are highly heterogeneous and may differ substantially between male and female patients. Being aware of these differences and performing preprocedural vascular mapping by CTA prior to surgical and interventional procedures have become critical prerequisites for optimizing treatment outcomes, preventing potential complications, and reducing morbidity and mortality. However, the statistically significant differences identified in this study were not observed across all evaluated vascular elements; therefore, these findings should be interpreted with caution and in a nuanced manner to ensure scientific accuracy.

## Figures and Tables

**Table 1:** Anatomical variations in celiac trunk and its branches.

Variable	*n* (%)
Sex	
Male	746 (63.8)
Female	424 (36.2)
Age mean ± sd [min–max]	60.54 ± 15.43 [21–92]
Variable	*n* (%)
Origin of the Celiac Trunk	
Classic celiac trunk	1092 (93.3)
Hepatosplenic trunk	28 (2.4)
Gastrosplenic trunk	38 (3.2)
Celiacomesenteric trunk	4 (0.3)
Absence of celiac trunk	4 (0.3)
Other	4 (0.3)
Origin of Celiac Trunk Branches	
Classic	1092 (93.6)
LGA arising directly from AA	8 (0.7)
LGA arising from SA	8 (0.7)
LGA arising from CHA/PHA	4 (0.3)
CHA arising directly from AA	9 (0.8)
Absence of CHA	18 (1.5)
Other (CHA originating from SMA)	8 (0.7)
Other (Common trunk of LHA + LGA)	4 (0.3)
Other (RHA from celiac trunk)	4 (0.3)
Other (LGA from AA + Absence of CHA)	4 (0.3)
Other (Splenic artery from SMA)	4 (0.3)
Other (Complex variation)	4 (0.3)

sd = standard deviation; *n* = number; min = minimum; max = maximum; LGA = left gastric artery; AA = abdominal aorta; SA = splenic artery; CHA = common hepatic artery; PHA = proper hepatic artery; SMA = superior mesenteric artery; LHA = left hepatic artery; RHA = right hepatic artery.

**Table 2:** Anatomical variations in IMA, SMA and its branches.

Variable	*n* (%)
Sex	
Male	746 (63.8)
Female	424 (36.2)
Age mean ± sd [min–max]	60.54 ± 15.43 [21–92]
Variable	*n* (%)
Origin of the SMA	
Classic	1136 (97.1)
Celiacomesenteric trunk	4 (0.3)
Hepatomesenteric trunk	20 (1.7)
Bimesenteric trunk	6 (0.5)
Other (Splenomesenteric)	4 (0.3)
Variations in SMA Branches	
Replaced RHA (dexter)	117 (10.0)
Replaced CHA	8 (0.7)
Replaced LHA	8 (0.7)
Other (Accessory RHA)	4 (0.3)
Other (Splenomesenteric)	4 (0.3)
No additional branch	1029 (87.9)
Origin of the IMA	
Classic	1152 (98.5)
Bimesenteric trunk	6 (0.5)
Other (Originating from SMA)	4 (0.3)
Indistinguishable	8 (0.7)

sd = standard deviation; *n* = number; min = minimum; max = maximum; CHA = common hepatic artery; SMA = superior mesenteric artery; LHA = left hepatic artery; RHA = right hepatic artery; IMA = inferior mesenteric artery.

**Table 3:** Anatomical variations in kidney, number of renal artery, hepatic arteries.

Variable	*n* (%)
Sex	
Male	746 (63.8)
Female	424 (36.2)
Age mean ± sd [min–max]	60.54 ± 15.43 [21–92]
Variable	*n* (%)
Associated Renal Anomaly	
Rotational anomaly	12 (1.0)
Horseshoe kidney	4 (0.3)
None	1154 (98.6)
Right Hepatic Artery (RHA)	
Classic	1004 (85.8)
Classic (originating from SMA)	4 (0.3)
Replaced (from GDA)	4 (0.3)
Replaced (from Celiac Trunk)	4 (0.3)
Replaced (from LHA)	4 (0.3)
Replaced (from SMA)	114 (9.7)
Accessory (from Aorta)	4 (0.3)
Accessory (from Celiac Trunk)	8 (0.7)
Accessory (from LHA)	6 (0.5)
Accessory (from SMA)	18 (1.5)
Median Number of Right Renal Arteries (IQR; min–max)	1 (1-1; 1-3) *
Left Hepatic Artery (LHA)	
Classic	1043 (89.1)
Replaced (from LGA)	64 (5.5)
Accessory (from LGA)	55 (4.7)
Replaced (from Celiac Trunk)	4 (0.3)
Replaced (from SMA)	4 (0.3)
Median Number of Left Renal Arteries (IQR; min–max)	1 (1-1; 1-3) *

* *p* value was obtained from Mann–Whitney U test; sd = standard deviation; *n* = number; LGA = left gastric artery; SMA = superior mesenteric artery; LHA = left hepatic artery; RHA = right hepatic artery; GDA = gastroduodenal artery; IQR = Interquartile Range; min = minimum; max = maximum.

**Table 4:** Comparison of celiac trunk and its branches between male and female patients.

Variable	Male *n* (%)	Female *n* (%)	*p*
Origin of the Celiac Trunk	746 (63.8)	424 (36.2)	
Classic celiac trunk	684 (91.7)	408 (96.2)	0.028 *
Hepatosplenic trunk	20 (2.7)	8 (1.9)	
Gastrosplenic trunk	30 (4.0)	8 (1.9)	
Celiacomesenteric trunk	4 (0.5)	0 (0.0)	
Absence of celiac trunk	4 (0.5)	0 (0.0)	
Other	4 (0.5)	0 (0.0)	
Origin of Celiac Trunk Branches	746 (63.8)	424 (36.2)	
Classic	684 (92.1)	408 (96.2)	<0.001 *
LGA arising directly from AA	4 (0.5)	4 (0.9)	
LGA arising from SA	9 (1.1)	0 (0.0)	
LGA arising from CHA/PHA	0 (0.0)	4 (0.9)	
CHA arising directly from AA	10 (1.2)	0 (0.0)	
Absence of CHA	19 (2.4)	0 (0.0)	
Other (CHA originating from SMA)	0 (0.0)	8 (1.9)	
Other (Common trunk of LHA + LGA)	4 (0.5)	0 (0.0)	
Other (RHA from celiac trunk)	4 (0.5)	0 (0.0)	
Other (LGA from AA + Absence of CHA)	4 (0.5)	0 (0.0)	
Other (Splenic artery from SMA)	4 (0.5)	0 (0.0)	
Other (Complex variation)	4 (0.5)	0 (0.0)	

* *p* value was obtained from Fisher–Freeman–Halton Exact Test. *n* = number; LGA = left gastric artery; AA = abdominal aorta; SA = splenic artery; CHA = common hepatic artery; PHA = proper hepatic artery; SMA = superior mesenteric artery; LHA = left hepatic artery; RHA = right hepatic artery.

**Table 5:** Comparison of SMA and IMA variations between male and female patients.

Variable	Male *n* (%)	Female *n* (%)	*p*
Origin of the SMA	746 (63.8)	424 (36.2)	
Classic	720 (96.5)	416 (98.1)	0.100 *
Celiacomesenteric trunk	4 (0.5)	0 (0.0)	
Hepatomesenteric trunk	12 (1.6)	8 (1.9)	
Bimesenteric trunk	6 (0.8)	0 (0.0)	
Other (Splenomesenteric)	4 (0.5)	0 (0.0)	
Variations in SMA Branches	746 (63.8)	424 (36.2)	
Replaced RHA	97 (13.0)	20 (4.7)	<0.001 *
Replaced CHA	4 (0.5)	4 (0.9)	
Replaced LHA	4 (0.5)	4 (0.9)	
Other (Accessory RHA)	4 (0.5)	0 (0.0)	
Other (Splenomesenteric)	4 (0.5)	0 (0.0)	
No additional branch	633 (84.9)	396 (93.4)	
Origin of the IMA	746 (63.8)	424 (36.2)	
Classic	732 (98.1)	420 (99.1)	<0.001 *
Bimesenteric trunk	6 (0.8)	0 (0.0)	
Other (Originating from SMA)	0 (0.0)	4 (0.9)	
Indistinguishable	8 (1.1)	0 (0.0)	

* *p* value was obtained from Fisher–Freeman–Halton Exact Test.; *n* = number; CHA = common hepatic artery; SMA = superior mesenteric artery; LHA = left hepatic artery; RHA = right hepatic artery; IMA = inferior mesenteric artery.

**Table 6:** Comparison of renal and hepatic variations between male and female patients.

Variable	Male *n* (%)	Female *n* (%)	*p*
Associated Renal Anomaly	746 (63.8)	424 (36.2)	
Rotational anomaly	0 (0.0)	12 (2.8)	<0.001 *
Horseshoe kidney	4 (0.5)	0 (0.0)	
None	742 (99.5)	412 (97.2)	
Right Hepatic Artery (RHA)	746 (63.8)	424 (36.2)	
Classic	616 (82.6)	388 (91.5)	<0.001 *
Classic (from SMA)	0 (0.0)	4 (0.9)	
Replaced (from GDA)	0 (0.0)	4 (0.9)	
Replaced (from Celiac Trunk)	4 (0.5)	0 (0.0)	
Replaced (from LHA)	0 (0.0)	4 (0.9)	
Replaced (from SMA)	98 (13.1)	16 (3.8)	
Accessory (from Aorta)	4 (0.5)	0 (0.0)	
Accessory (from Celiac Trunk)	8 (1.1)	0 (0.0)	
Accessory (from LHA)	6 (0.8)	0 (0.0)	
Accessory (from SMA)	10 (1.3)	8 (1.9)	
Median Number of RAA (IQR; min–max)	1(1-1; 1-3)	1(1-1; 1-3)	0.078 **
Left Hepatic Artery (LHA)	746 (63.8)	424 (36.2)	
Classic	637 (85.4)	406 (95.8)	<0.001 *
Replaced (from LGA)	60 (8.0)	4 (0.9)	
Accessory (from LGA)	41 (5.5)	14 (3.3)	
Replaced (from Celiac Trunk)	4 (0.5)	0 (0.0)	
Replaced (from SMA)	4 (0.5)	0 (0.0)	
Median Number of LRA (IQR; min–max)	1(1-1; 1-3)	1(1-1; 1-3)	0.093 **

* *p* value was obtained from Fisher–Freeman–Halton Exact Test ** *p* value was obtained from Mann–Whitney U test; *n* = number; LGA = left gastric artery; SMA = superior mesenteric artery; LHA = left hepatic artery; RHA = right hepatic artery; GDA = gastroduodenal artery; RAA = right renal artery; LRA: left renal artery; IQR = Interquartile Range; min = minimum; max = maximum.

## Data Availability

Data available on request due to restrictions (legal reasons).

## Abbreviations

The following abbreviations are used in this manuscript:

MDCT	Multidetector Computed Tomography
TACE	Transcatheter arterial chemoembolization
TARE	Transcatheter arterial radioembolization
CTA	MDCT angiography
ROI	Region of Interest
HU	Hounsfield Unit
sd	Standard deviation
*n*	Number
LGA	Left gastric artery
AA	Abdominal aorta
SA	Splenic artery
CHA	Common hepatic artery
PHA	Proper hepatic artery
SMA	Superior mesenteric artery
LHA	Left hepatic artery
RHA	Right hepatic artery
IMA	Inferior mesenteric artery
GDA	Gastroduodenal artery
min	Minimum
max	Maximum
